# Impact of viral load on sample pooling for reverse-transcription polymerase chain reaction detection-based diagnosis of coronavirus disease 2019 in Nigeria

**DOI:** 10.4102/ajlm.v14i1.2514

**Published:** 2025-02-10

**Authors:** Timan T. Eliya, Elvis E. Isere, Bassey Emmana, Chukwuebuka Ugwu, Jonathan Kushim, Precious Ishaku, Aisha E. Ibrahim, John S. Bimba

**Affiliations:** 1 Zankli Research Centre, Bingham University, Karu, Nasarawa State, Nigeria; 2 Department of Biological Sciences, Faculty of Sciences, Bingham University, Karu, Nasarawa State, Nigeria; 3 Department of Community Medicine, Bingham University, Karu, Nasarawa State, Nigeria; 4 Department of Clinical Sciences, Liverpool School of Tropical Medicine, Liverpool, United Kingdom

**Keywords:** coronavirus disease 2019, pandemic, real-time polymerase chain reaction testing, cycle threshold, sample pooling, Nigeria

## Abstract

**Background:**

The coronavirus disease 2019 (COVID-19) pandemic strained diagnostic testing capacities globally, particularly in low- and middle-income countries like Nigeria. Reverse-transcription polymerase chain reaction (RT-PCR) remains the gold standard for COVID-19 detection, but limited testing resources caused bottlenecks in Nigeria’s response during the pandemic. Sample pooling offers a cost-effective strategy to enhance testing capacity during future outbreaks.

**Objective:**

This study determined the maximum number of COVID-19 samples that can be pooled for RT-PCR testing in Nigeria without compromising the detection sensitivity of a single positive sample.

**Methods:**

A total of 1222 nasopharyngeal samples from symptomatic COVID-19 patients in Nasarawa State, Nigeria, collected between March 2021 and August 2022, were retrieved from the laboratory biorepository and analysed from November 2022 to February 2023. These included five positive samples with cycle threshold (Ct) values ranging from ≤ 20 to 40, and 1217 negative samples. Positive samples were pooled with negative ones at increasing dilution ratios (1:4–1:64), to assess detection sensitivity on the GeneXpert platform.

**Results:**

A positive sample with a Ct value ≤ 25 could be pooled with up to 64 negative samples while maintaining a detectable positive result. However, samples with Ct values of 36–40 could only be pooled with a maximum of eight negative samples. Higher Ct values reduced pooling effectiveness.

**Conclusion:**

Sample pooling is a feasible method for scaling up COVID-19 RT-PCR testing in resource-limited settings like Nigeria. The Ct value is critical in determining optimal pool sizes for accurate detection.

**What this study adds:**

The findings provide critical guidelines for determining the optimal pool sizes based on Ct values, aiding in effective COVID-19 testing strategies. By optimising sample pooling based on viral load, health authorities can improve their response to future COVID-19 outbreaks and similar public health emergencies.

## Introduction

The coronavirus disease 2019 (COVID-19) pandemic, caused by severe acute respiratory syndrome coronavirus 2, created a global health crisis and drastically increased the demand for diagnostic testing.^1,2^ On 28 February 2020, the Nigeria Centre for Disease Control and Prevention confirmed the first COVID-19 case in Nigeria.^3,4^ This was followed by rising daily cases and deaths across several states and the Federal Capital Territory, including Nasarawa State, which indicated widespread community transmission and unmet testing needs.^5,6^

**FIGURE 1:**
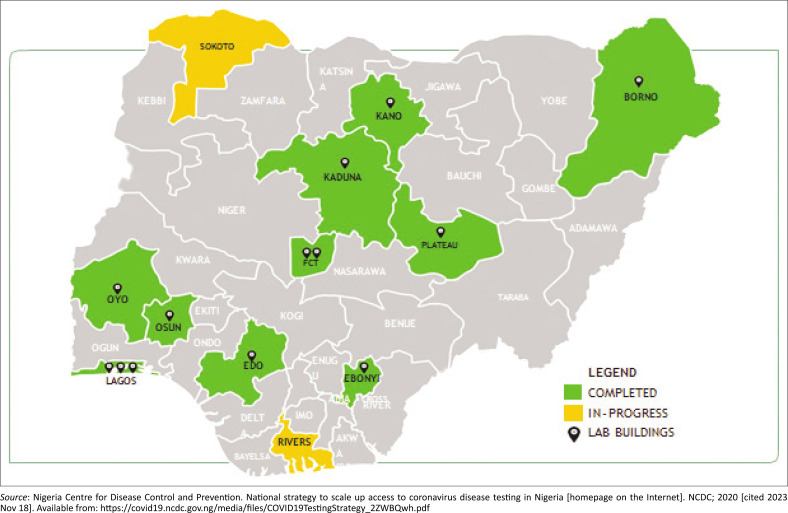
Laboratory testing capacity for coronavirus disease 2019 in Nigeria as of 15 April 2020.

As with many low- and middle-income countries, Nigeria faced significant challenges in diagnostic testing during the pandemic because of limited resources. Reverse-transcription polymerase chain reaction (RT-PCR), the gold standard for COVID-19 diagnosis, was constrained by shortages of kits, reagents, and trained personnel.^3^ As of April 2020, Nigeria had only 12 functional RT-PCR facilities (Figure 1),^3^ which increased to 97 by January 2021, but these remained insufficient for a population of over 250 million.^3,7^ These limitations caused testing delays, hindered contact tracing, and underreported cases, weakening Nigeria’s pandemic response.^8^

Sample pooling, a cost-effective diagnostic technique in which multiple samples were combined and tested together, emerged as a potential solution to these challenges.^8,9,10,11,12,13,14,15,16,17,18,19^ In this method, multiple patient samples were tested together as a single pool.^14,15,16^ If the pool tested negative, all samples were considered negative; if positive, each sample was tested individually to identify which samples were positive.^17,18^ This method reduced the number of tests required, conserved resources, reduced laboratory workload, and increased testing capacity.^8,10,11^ In countries like Ghana, sample pooling proved successful during the pandemic.^8,10,11^ However, the effectiveness of pooling depended on several factors, including viral load, and determining the optimal number of samples per pool was crucial for ensuring PCR accuracy and efficiency.^19^

Despite the global decline in COVID-19 cases, as well as in Nigeria, the threat of future pandemics and outbreaks due to COVID-19 remains, especially with the emergence of different COVID-19 variants, highlighting the importance of strengthening diagnostic systems.^7^ Assessing the efficiency of sample pooling in Nigeria, which was not widely implemented during the pandemic, could be crucial in preparedness for future outbreak response. This study aimed to determine the optimal number of COVID-19 samples that could be pooled without missing a positive result in the Nigeria setting, in order to enhance testing efficiency during any future COVID-19 outbreak response in Nigeria.

## Methods

### Ethical considerations

Ethical approval for this study was obtained from the Federal Capital Territory Health Research Ethics Committee (reference no: FHREC/2024/01/48/04-03-24). A waiver for informed patient consent was granted, as stored laboratory samples were retrieved solely for analysis, with no additional clinical procedures involving patients. All personal information and patient identifiers were removed from the samples and study-specific numbers (identifiers) were assigned to each. These identifiers and outcome of the laboratory analysis were entered into an anonymised, password-protected Excel spreadsheet (Office 2016, Microsoft, Redmond, Washington, United States), accessible only to the study investigators for data analysis.

### Study area

Nigeria, the most populous country in Africa, with over 250 million people,^20^ is organised into six distinct regional zones, each with unique ecological, climatic, and demographic characteristics.^20,21^ Administratively, the country is divided into 36 states plus the Federal Capital Territory, comprising 774 local government areas and 8812 wards.^21^ Nasarawa State, situated in the North Central zone, had a projected population of 3 079 710 in 2023.^22^ This state features a healthcare system of 1040 facilities, including 728 primary health centres, 18 secondary hospitals, 2 tertiary hospitals, and 292 private facilities.^20,23^

### Study specimens and laboratory analysis

The samples for this study were aliquots stored in cryovials, retrieved from the Zankli Research Centre Molecular Laboratory biorepository at Bingham University, Nasarawa State, Nigeria. These included nasopharyngeal swab specimens in viral transport medium (MANTACC Viral Transport Media disposable sampling kit; Huo-Yan Laboratory, Shenzhen, China), collected from symptomatic patients suspected of COVID-19 infection across communities in Nasarawa State, Nigeria, between March 2021 and August 2022. Initially tested for COVID-19 following Nigeria Centre for Disease Control and Prevention guidelines and procedures,^24^ the samples were subsequently stored in an ultra-low freezer at –85 °C. The samples retrieved for this study comprised five positive samples and 1217 negative samples, each with documented cycle threshold (Ct) values.

The retrieved samples were analysed between November 2022 and February 2023. A repeat RT-PCR test was conducted to re-confirm the Ct values for each sample before performing the pooled test procedure. These positive samples were tested individually by RT-PCR on Cepheid’s GeneXpert^®^ systems with the Xpert^®^ Xpress SARS-CoV-2 (Cepheid, Sunnyvale, California, United States), and their Ct values were grouped in the following ranges: ≤ 20, 21 ‒25, 26 ‒ 30, 31 ‒ 35, and 36 ‒ 40, to represent a decreasing viral load, with ‘≤ 20’ representing the highest and ’36‒40’ representing the lowest. Similarly, 1217 samples confirmed to be negative through RT-PCR were pooled to create different pools of varying dilution factors.

All procedures were conducted in a NuAire Class II 12469:2000 biological safety cabinet within the containment laboratory room by trained personnel. Samples and pools were tested using Cepheid’s GeneXpert^®^ systems following the manufacturer’s instructions. Each selected positive sample was pooled with negative samples at increasing dilution factors of 1:4 until the Ct value of the pool with the highest dilution factor exceeded 40 while remaining positive. The Ct values of each pool for each positive sample were recorded.

For the first pool (1:4), 70 µL of the positive sample and 70 µL of each of the four (4) negative samples were transferred to a cryovial to obtain the 1:4 dilution factors. The contents were vortexed, transferred to a GeneXpert cartridge and loaded onto the GeneXpert platform according to the manufacturer’s instructions. The same procedure was used to constitute pools of 1:8, up through 1:64 for each of the five (5) positive samples.

### Data management and analysis

The Ct values of the test outcomes from the RT-PCR analysis of pooled samples were entered into an anonymised, password-protected Excel spreadsheet (Office 2016, Microsoft, Redmond, Washington, United States), and the data were analysed descriptively using charts and tables. Samples with Ct values ≤ 43 were considered positive for COVID-19 infection, while samples with Ct values > 43 were considered negative for COVID-19 infection.

## Results

**FIGURE 2:**
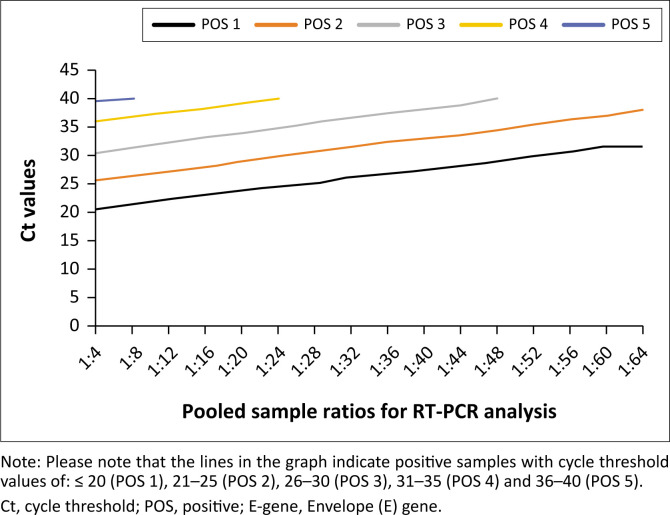
Distribution of cycle threshold values of a single positive sample across different pools of varying numbers of negative samples in Nasarawa State, Nigeria, from November 2022 to February 2023.

As shown in Table 1 and Figure 2, our investigation revealed that a single positive COVID-19 sample with Ct values ≤ 20 and 21–25 could be pooled with as many as 63 other samples to obtain a positive pool, which signifies that such a single positive sample is detectable in a pool of up to 1:64 negative samples. However, pooling COVID-19 samples with a single positive COVID-19 sample with Ct values 26–30 can only be detected in a pool of 1:48 negative samples using RT-PCR, 31–35 as 1:24, and 36–40 as 1:8 (Table 1 and Figure 2).

**TABLE 1:** Positivity across different pools of varying dilution factors based on the cycle threshold value of a single positive sample among 4 to 64 negative samples in Nasarawa State, Nigeria, from November 2022 to February 2023.

Positive Ct value	Cycle threshold values for pools of varying dilution factors															
	1:4	1:8	1:12	1:16	1:20	1:24	1:28	1:32	1:36	1:40	1:44	1:48	1:52	1:56	1:60	1:64
≤ 20	20.6	21.7	22.3	23.3	23.8	24.7	25.5	26.4	26.9	27.5	28.3	29.2	30.1	30.8	31.7	31.7
21–25	25.5	26.5	27.4	23.3	29.3	30.0	30.8	31.5	32.6	33.2	33.9	34.6	35.8	36.5	37.3	38.2
26–30	30.8	31.5	32.6	23.3	34.2	35.0	35.9	36.8	37.6	38.4	39.2	40.2	44.2	44.2	44.2	44.2
31–35	36.2	37.2	38.0	23.3	39.3	40.2	44.2	44.2	44.2	44.2	44.2	44.2	44.2	44.2	44.2	44.2
36–40	39.6	40.3	44.2	44.2	44.2	44.2	44.2	44.2	44.2	44.2	44.2	44.2	44.2	44.2	44.2	44.2

Note: Cycle threshold values ≥ 43.0 signifies a negative test result.

Ct, cycle threshold.

## Discussion

This investigation showed that the Ct value was a determining factor in choosing the maximum number of samples to be pooled for effective RT-PCR detection of COVID-19. However, determining the maximum number of samples that can be pooled while still maintaining reliable and accurate test results is a critical challenge to consider.

In our study, we discovered that a single positive sample with a presumed Ct value of ≤ 25 could be pooled with up to 64 samples to obtain a positive pool, while a single presumed positive sample with a Ct value > 40 could only be pooled with a maximum of 8 presumed negative samples to obtain a positive pool. A similar study carried out in Israel in 2020 reported that using a pooled testing approach for standard RT-PCR for COVID-19 detection with a single positive sample could detect a pool of up to 32 samples, with an estimated false-negative rate of 10%.^20^

A study from Malaysia, evaluating the effectiveness of pooled sample testing for COVID-19 RT-PCR, conducted in 2020 by Lim et al.,^1^ utilised both retrospective and prospective samples. The findings indicated that pooled testing using volumes of 25 µL, 40 µL, 60 µL, and 100 µL produced test results comparable to individual testing, with positive cases detected consistently across pool sizes. The study defined a positive result as having a Ct value of 38 or lower, as recommended in the World Health Organization-Charité protocol.^1^ This finding is consistent with our research outcome, where a single positive sample with a Ct value > 40 could only be pooled with a maximum of 8 negative samples to obtain a positive pool. Our study revealed that the detection sensitivity of this method was similar to that of testing individual samples alone.

Similarly, Wacharapluesadee et al.,^25^ in a 2020 study in Thailand, found that pooling specimens did not reduce the sensitivity of detecting severe acute respiratory syndrome coronavirus 2 when the original specimen had a Ct value below 35. However, for specimens with low viral loads (Ct > 35), 13.3% (2 out of 15 pools) produced false-negative results. Therefore, a high Ct value indicates that pooling is not suitable for testing, and it is recommended to reduce the number of samples in the pool.^25^

The Ct value is a measure of the amount of RT-PCR amplification required to detect a signal from the target nucleic acid in a sample.^26^ The Ct value is inversely proportional to the amount of target nucleic acid present in the sample. The higher the Ct value, the lower the amount of target nucleic acid in the sample.^26^ A Ct value is considered positive for COVID-19 when it is detectable within the test’s threshold, while undetectable values indicate a negative result for that test, as they fall below the test’s sensitivity range.^26^

Notably, the maximum number of samples that can be pooled while still maintaining accurate and reliable results may vary depending on the prevalence of COVID-19 in the population being tested, the assay used, and the quality of the samples.^15,27,28,29,30,31^ If the prevalence of COVID-19 in the population is low, it may be necessary to reduce the number of samples in the pool to maintain accurate and reliable results; likewise, if the quality of the samples is poor, it may be necessary to reduce the number of samples in the pool to ensure accurate results.^15,27,28,29^

Most importantly, determining the optimal pool size for RT-PCR testing for COVID-19 detection requires consideration of several factors, including the presumed viral load of positive samples, the Ct value, the sensitivity of the RT-PCR test, the prevalence of COVID-19 in the population, and the cost and time efficiency of the testing process.^7,15,27,28,29^ While different studies have recommended different pool sizes, there is no one-size-fits-all approach, and the optimal pool size may vary depending on the context and resources available. Therefore, a process of trial and error was necessary to determine the optimal pool size for RT-PCR testing in specific settings.^15^ However, this study provided a more detailed number of samples that can effectively be pooled for greater sensitivity in relation to the presumed Ct values of the positive samples among the preceding batches of samples from the same location and period as shown Table 1.

Additionally, Yani et al.,^32^ in a study comparing RT-PCR Ct values between individual and pooled severe acute respiratory syndrome coronavirus 2-infected nasopharyngeal swab specimens, conducted in Indonesia in 2024, had reported no difference in the Ct values between individual sample and pooled sample groups at all concentrations and for all pooled sizes, emphasising that a pooled RT-PCR testing strategy did not reduce the quality of individually measured RT-PCR Ct values.^32^

### Limitations

A limitation of this study is that we did not collect information on clinical data or symptoms; we believe that all patients may have been symptomatic with COVID-19-related symptoms, as stated in the study protocol, as this was strictly monitored to eliminate protocol deviations.

Additionally, RT-PCR, like most other testing systems, could have misclassified some negative samples as positive and vice versa during the evaluation cycle. These errors might result from improper sample handling, deficient sample loading, or RNA degradation during processing. However, to minimise such errors, we ensured strict adherence to all standard operating procedures for laboratory practices under the supervision of highly experienced senior laboratory scientists throughout the study implementation

### Conclusion

Sample pooling for RT-PCR of COVID-19 samples is a promising strategy that could increase testing capacity and overcome the limitations of current testing methods during a COVID‒19 pandemic, especially in Nigeria, which has a limited capacity for RT-PCR. However, sample pooling requires careful planning, coordination, and optimisation to ensure that it is both cost-effective and reliable. This research revealed that the presumed Ct value is a determining factor in choosing the maximum number of samples to be pooled for effective RT-PCR detection of COVID-19, and provides a guide for COVID-19 sample pooling in Nigeria for current and future outbreak responses.
